# Targeting ERK1/2-bim signaling cascades by BH3-mimetic ABT-737 as an alternative therapeutic strategy for oral cancer

**DOI:** 10.18632/oncotarget.5523

**Published:** 2015-10-02

**Authors:** Ji-Ae Shin, Lee-Han Kim, Sook-Jeong Lee, Joseph H. Jeong, Ji-Youn Jung, Hae Nim Lee, In-Sun Hong, Sung-Dae Cho

**Affiliations:** ^1^ Department of Oral Pathology, School of Dentistry, Institute of Biodegradable Material, Institute of Oral Bioscience, Chonbuk National University, Jeonju, Republic of Korea; ^2^ Department of New Drug Discovery and Development, Chungnam National University, Daejon, Republic of Korea; ^3^ Department of Dermatology and Biochemistry, Medical College of Wisconsin, Milwaukee, Wisconsin, USA; ^4^ Department of Companion and Laboratory Animal Science, Kongju National University, Yesan, Republic of Korea; ^5^ Department of Molecular Medicine, School of Medicine, Lee Gil Ya Cancer and Diabetes Institute, Gachon University, Incheon, Republic of Korea

**Keywords:** BH3 mimetics, ABT-737, oral cancer, apoptosis, Bcl-2 family

## Abstract

To date, many different chemotherapeutic agents have been widely used as common treatments for oral cancers. However, their therapeutic effects have been disappointing, and these agents may have unwanted side effects. Among the many regulatory factors, overexpression of pro-survival Bcl-2 family members may promote resistance to chemotherapeutic drugs in many tumors. The BH3 domain-only proteins effectively antagonize their apoptotic activities. Therefore, there is substantial interest in developing chemotherapeutic drugs that directly target pro-survival Bcl-2 proteins by mimicking the BH3 domain and unleashing pro-apoptotic molecules in tumor cells. Among the numerous available small molecule BH3 mimetics, ABT-737, a potent small molecule that binds to Bcl-2/Bcl-xL with high affinity, has anti-tumor activity in a wide variety of cancer cells. However, the effects of ABT-737 on human oral cancers and the underlying molecular mechanisms have not previously been elucidated. In the present study, we observed that inactivation of the ERK1/2 signaling pathway using ABT-737 dramatically increased the expression of pro-apoptotic protein Bim via transcriptional and/or posttranslational regulation, in a cell type-dependent manner, inducing mitochondria-mediated apoptosis of human oral cancer cells. To the best of our knowledge, this is the first demonstration of the antitumor effects of ABT-737 on human oral cancers.

## INTRODUCTION

Apoptosis is a natural barrier that prevents cancer cells from surviving and disseminating to other tissues. However, cancer cells use various strategies to avoid apoptotic cell death by modulating multiple key regulators of the apoptotic signaling pathway. Apoptosis evasion contributes to therapeutic resistance to conventional chemotherapy and accounts for the so-called ‘poor responders’ [1]. Therefore, there have been recent attempts to develop effective anticancer strategies targeting apoptotic signaling pathways [2], such as through inhibiting anti-apoptotic survival factors, which are likely overexpressed in many tumors and may confer drug-resistant phenotypes [2, 3]. Among these factors, pro-survival Bcl-2 family proteins have attracted considerable attention. The family, a group of structurally related proteins, consists of the pro-apoptotic members Bax and Bak, which contain the Bcl-2 Homology 3 (BH3) domain, and pro-survival members Bcl-B, Bcl-xL, Bcl-w, Bcl-2, Bfl-1, and Mcl-1 [4, 5]. Overexpression of pro-survival Bcl-2 family members may drive tumorigenesis and resistance to chemotherapeutic drugs in many tumors [4, 5]. Suppression of pro-survival Bcl-2 family proteins with antisense oligonucleotides [6] or siRNA [7] has been proposed to restrict their functions, but their application *in vivo* remains problematic due to their relatively poor stability, bioavailability, and rapid modification in bio-fluids.

The activities of anti-apoptotic Bcl-2 family proteins are effectively antagonized by the “BH3 domain-only” proteins (i.e., Bad and Bim). The BH3-only proteins can bind to and neutralize pro-survival Bcl-2 proteins, resulting in the release and activation of pro-apoptotic Bak and/or Bax. Therefore, there is substantial interest in developing potential chemotherapeutic drugs that directly target pro-survival Bcl-2 proteins by mimicking the BH3 domain to unleash pro-apoptotic molecules in tumor cells [8–10]. These mimetics selectively and effectively attack cancer cells, which are most likely because many cancer cell types are primed for apoptotic cell death [11]. It has been postulated that rapidly growing cancer cells tend to activate the Bak/Bax pro-apoptotic signaling pathway, the activation of which is counteracted by increased levels of pro-survival Bcl-2 members. BH3-mimetics selectively release Bak/Bax from their pro-survival Bcl-2-like counterparts and allow them to promote apoptotic cell death. Among the numerous available BH3 mimetics, the best-characterized molecule is probably ABT-737, which binds with high affinity (in the nmol/L range) to Bcl-2, Bcl-xL and Bcl-w and with weak affinity to Mcl-1 and BFL/A1 (1000-fold lower affinities) [12]. As a single agent, ABT-737 has high anti-tumor activity in a wide variety of cancer cells, such as lymphoma [13] and several solid tumor cell types [14, 15]. However, the effects of ABT-737 on human oral cancers and the underlying molecular mechanisms have never been elucidated.

Around 90 percent of oral cancers may arise in squamous cells called oral squamous cell carcinoma (OSCC). Mucoepidermoid carcinoma, which is the most common malignant tumor in salivary glands, is a less common cause of oral cancer but is more serious when present. Because oral cancer usually has low chemo sensitivity, the results are commonly disappointing and therapeutic effects are achievable in a only minority of patients [16] and are accompanied by serious side effects [17, 18]. Therefore, in the present study, we investigated, for the first time, the anti-cancer effect of ABT-737 in human squamous carcinoma and mucoepidermoid carcinoma cells. To the best of our knowledge, this is the first demonstration of the anti-cancer effects of ABT-737 human oral squamous carcinoma and mucoepidermoid carcinoma and cells, respectively. Moreover, we also found that the effects of ABT-737 on human oral cancer cells may be attributed to the regulation of Bim levels by modulation of the ERK1/2 signaling pathway in a cell type-dependent manner.

## RESULTS

### ABT-737 inhibits the growth of oral cancer cells by inducing apoptosis

**Figure 1:**
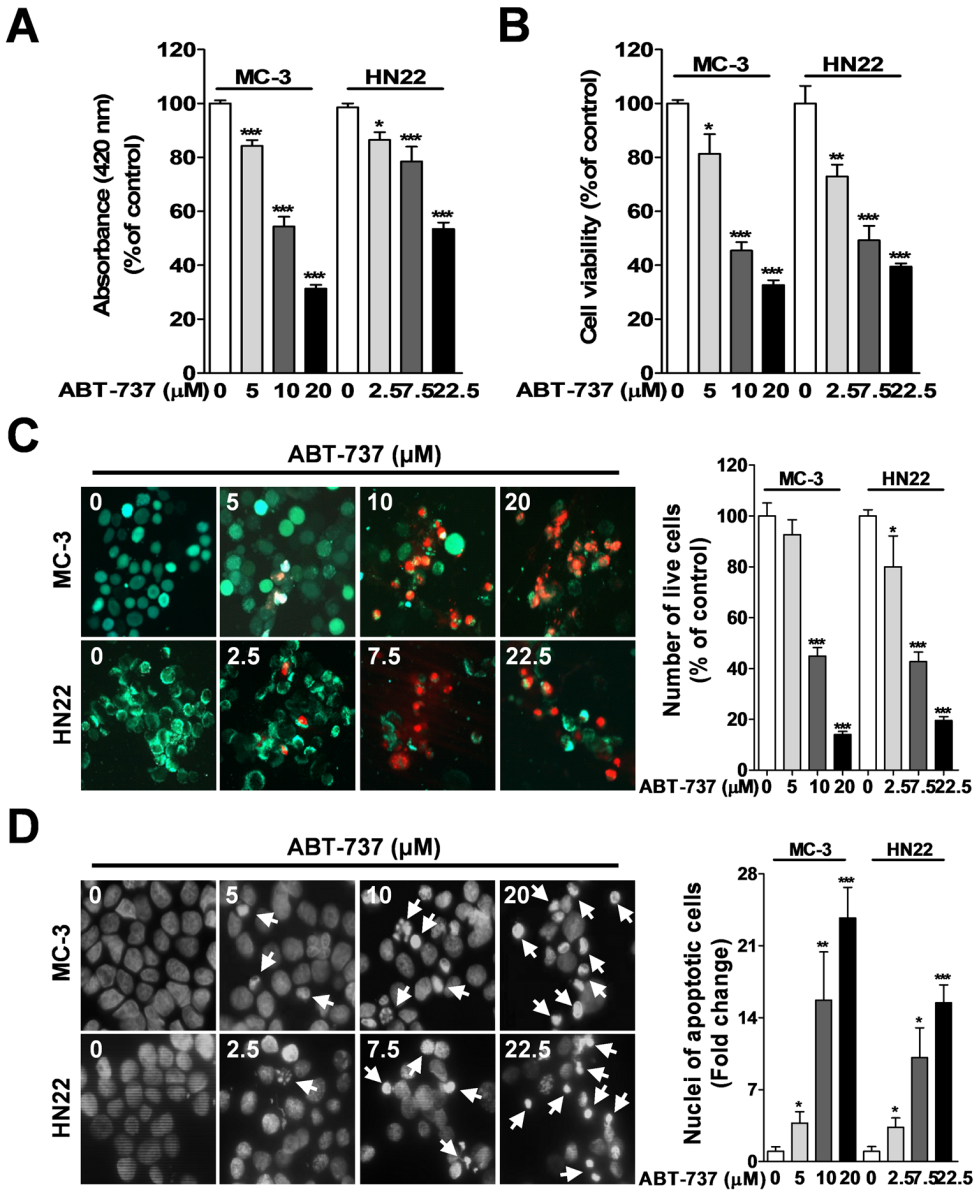
Effect of ABT-737 on cell viability and apoptosis in human oral cancer cells MC-3 and HN22 cells were treated with or without ABT-737 for 24 hr at different concentrations. Cell viability was evaluated using a MTS assay **A.** and trypan blue dye exclusion assay **B.** Qualitative estimation of ABT-737-induced apoptotic cell death was obtained by a live/dead assay, which differentially labels live (green) and dead (red) cells with fluorescent dyes **C.** With fluorescence microscopy (magnification X200), MC-3 and HN22 cells exhibited nuclear fragmentation and condensation, as determined by DAPI staining **D.** The results are presented as the mean ± SD from three independent experiments. *P < 0.05, **P < 0.01, and ***P < 0.001.

To assess the anti-proliferative potential of ABT-737 on human mucoepidermoid carcinoma (MC-3) and human oral squamous carcinoma (HN22) cells, both cell lines were treated with various concentrations of ABT-737. Cell viability was determined with the MTS (Figure 1A) and trypan blue dye exclusion assays (Figure 1B). ABT-737 inhibited MC-3 and HN22 cell growth in a concentration-dependent manner after 24 hr. Qualitative estimation of ABT-737-induced apoptotic cell death was obtained using the live/dead assay, which differentially labels live (green) and dead (red) cells with fluorescent dyes (Figure 1C). Apoptotic cell death was also qualitatively estimated by DAPI staining for nuclear condensation and fragmentation. ABT-737 treatment resulted in significant DNA fragmentation compared to untreated controls (Figure 1D). These results indicated that ABT-737 might induce apoptosis-mediated cell growth inhibition and offer a novel therapeutic agent to suppress human oral cancer cells.

### ABT-737 induces caspase-mediated apoptosis in human oral cancer cells

**Figure 2:**
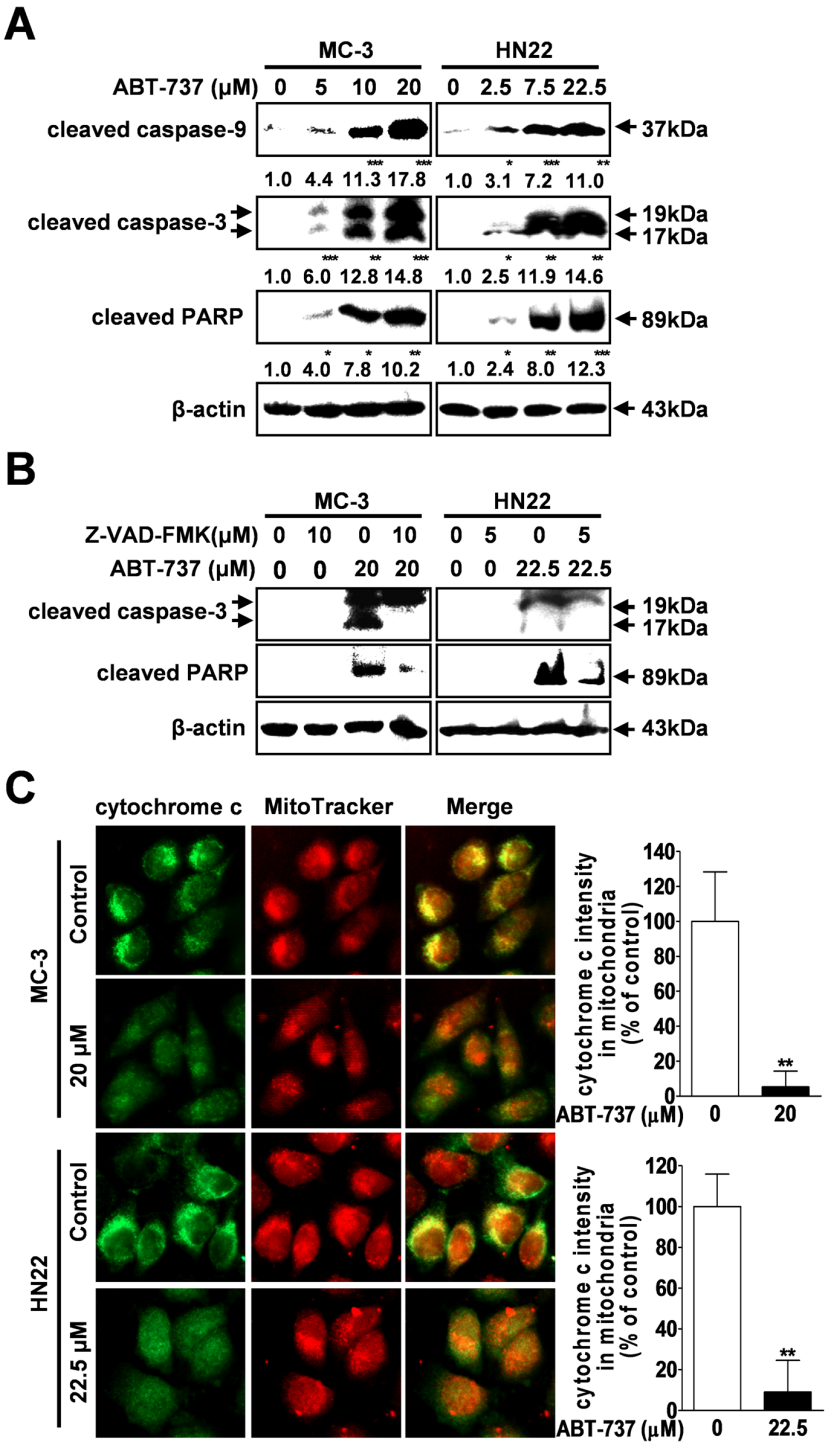
Effect of ABT-737 on caspase-mediated apoptosis in human oral cancer cells MC-3 and HN22 cells were treated with or without ABT-737, and the expression levels of cleaved caspase-9 and 3 and cleaved PARP were analyzed by Western blot analysis **A.** MC-3 and HN22 cells were preincubated with a pancaspase inhibitor (Z-VAD-FMK) for 1 hr prior to treatment with ABT-737; afterwards, cells were harvested for Western blot analysis to detect cleaved caspase-3 and cleaved PARP **B.** Immunocytochemistry revealed the release of mitochondrial cytochrome c (green) into the cytoplasm after ABT-737 exposure (20 μM in MC-3 cells and 22.5 μM in HN22 cells) for 24 hr. MitoTracker (red) staining labels mitochondria within each field **C.** β-actin was used as an internal control. The results are shown as the mean ± SD from three independent experiments. *P < 0.05, **P < 0.01, and ***P < 0.001.

Next, we investigated whether ABT-737-induced apoptotic cell death depends on the caspase-mediated pathway. Western blot results revealed that ABT-737 treatment induced the activation of initiator caspase (caspase-9), effector caspase (caspase-3), and PARP (substrate for caspase-3) in MC-3 and HN22 cells (Figure 2A). To further confirm whether ABT-737-induced cell death depends on caspase-mediated activation, MC-3 and HN22 cells were pre-incubated with the broad-spectrum caspase inhibitor Z-VAD-FMK before ABT-737 treatment. Pretreatment of cells with Z-VAD-FMK successfully attenuated ABT-737-induced caspase-3 and PARP cleavage, suggesting that ABT-737 induces caspase-dependent apoptosis in MC-3 and HN22 cells (Figure 2B). Accumulating evidence shows that pro-caspase-9 is cleaved into active caspase-9 in a cytochrome c-dependent manner [19]. Thus, mitochondrial cytochrome c release is a key step for the initiation of caspase 9-dependent apoptosis. In this context, we investigated whether increasing cytochrome c levels in cytosol might be the mechanism by which ABT-737 promotes apoptotic cell death. The subcellular localization of cytochrome c after ABT-737 treatment induction was studied using the fluorescent dye MitoTracker, which accumulates in mitochondria. Treatment of cells with ABT-737 resulted in the release of mitochondrial cytochrome c into the cytosol, which is where it mediates caspase-dependent apoptosis (Figure 2C).

### ABT-737 induces mitochondria-mediated apoptosis through the pro-apoptotic Bcl-2 protein bax

**Figure 3:**
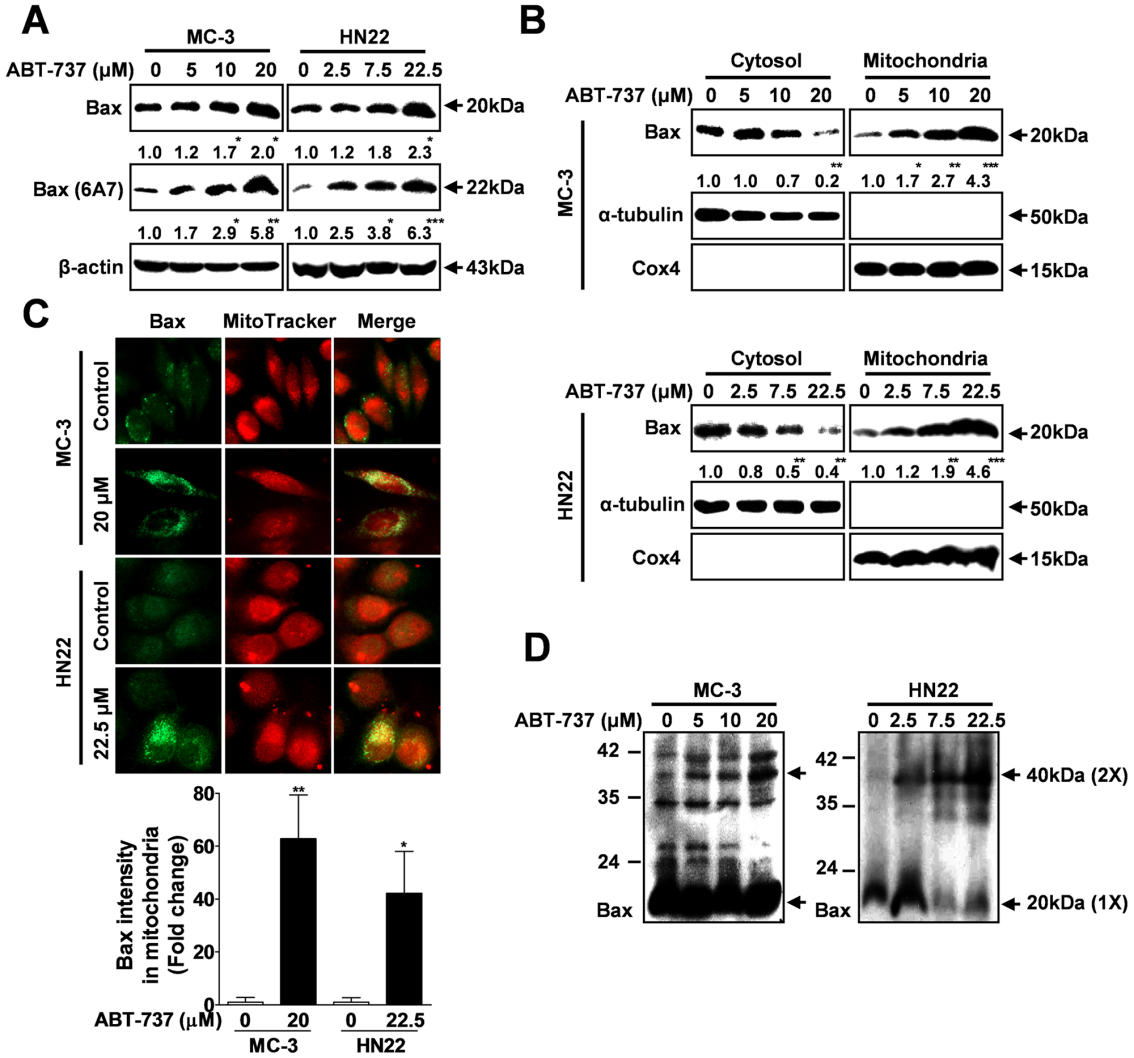
ABT-737 induces mitochondrial-dependent apoptosis through Bax MC-3 and HN22 cells were treated with or without ABT-737 for 24 hr, total cellular protein was prepared, and the protein levels of total and active Bax (6A7) were evaluated by Western blot analysis **A.** MC-3 and HN22 cells were treated with or without ABT-737 for 24 hr; afterwards, cytosolic and mitochondrial fractions were prepared and the protein levels of total and active Bax (6A7) were evaluated by Western blot analysis **B.** Immunocytochemistry revealed the mitochondrial translocation of Bax from the cytoplasm after ABT-737 treatment (20 μM in MC-3 cells and 22.5 μM in HN22 cells) for 24 hr. MitoTracker (red) staining labels mitochondria within each field **C.** To assess whether ABT-737 treatment has an effect on the dimerization of Bax, the levels of dimeric Bax were measured with or without ABT-737 treatment in MC-3 and HN22 cells **D.** β-actin was used as an internal control. The Cox4 exclusive mitochondria marker was used as a control for the separation of mitochondrial and cytosolic fractions. The results are shown as the mean ± SD from three independent experiments. *P < 0.05, **P < 0.01, and ***P < 0.001.

Exposure of an N-terminal epitope of Bax seems to induce apoptotic cell death by controlling this mitochondrial membrane permeability during apoptosis [20, 21]. Therefore, we examined whether ABT-737 treatment could affect the expression of pro-apoptotic protein Bax using the total Bax and active Bax (6A7) N-terminal-specific antibodies. The levels of the total and active Bax were significantly increased in both cell lines compared with the controls (Figure 3A). Furthermore, the pro-apoptotic proteins Bax, which normally reside in the cytoplasm of healthy cells, translocate to the mitochondrial outer membrane when triggered by apoptotic stimuli [22], where they cause cytochrome c release from the mitochondria [23]. Western blot analysis (Figure 3B) and an immunofluorescence assay (Figure 3C) revealed that there is mitochondrial translocation of Bax from the cytoplasm after ABT-737 treatment. Bax is present as a high molecular weight oligomer/complex in the apoptotic cells by causing permeabilization of the mitochondrial outer membrane [24]. Therefore, to further assess whether ABT-737 treatment affects Bax dimerization, we measured the levels of dimeric Bax with or without treatment. ABT-737 exposure significantly increased the level of dimeric Bax compared to negative controls (Figure 3D). Taken together, these results indicated that ABT-737 induced Bax-mediated apoptosis by increasing its translocation and dimerization.

### Bim may activate bax signaling to mediate ABT-737-induced apoptotic cell death

**Figure 4:**
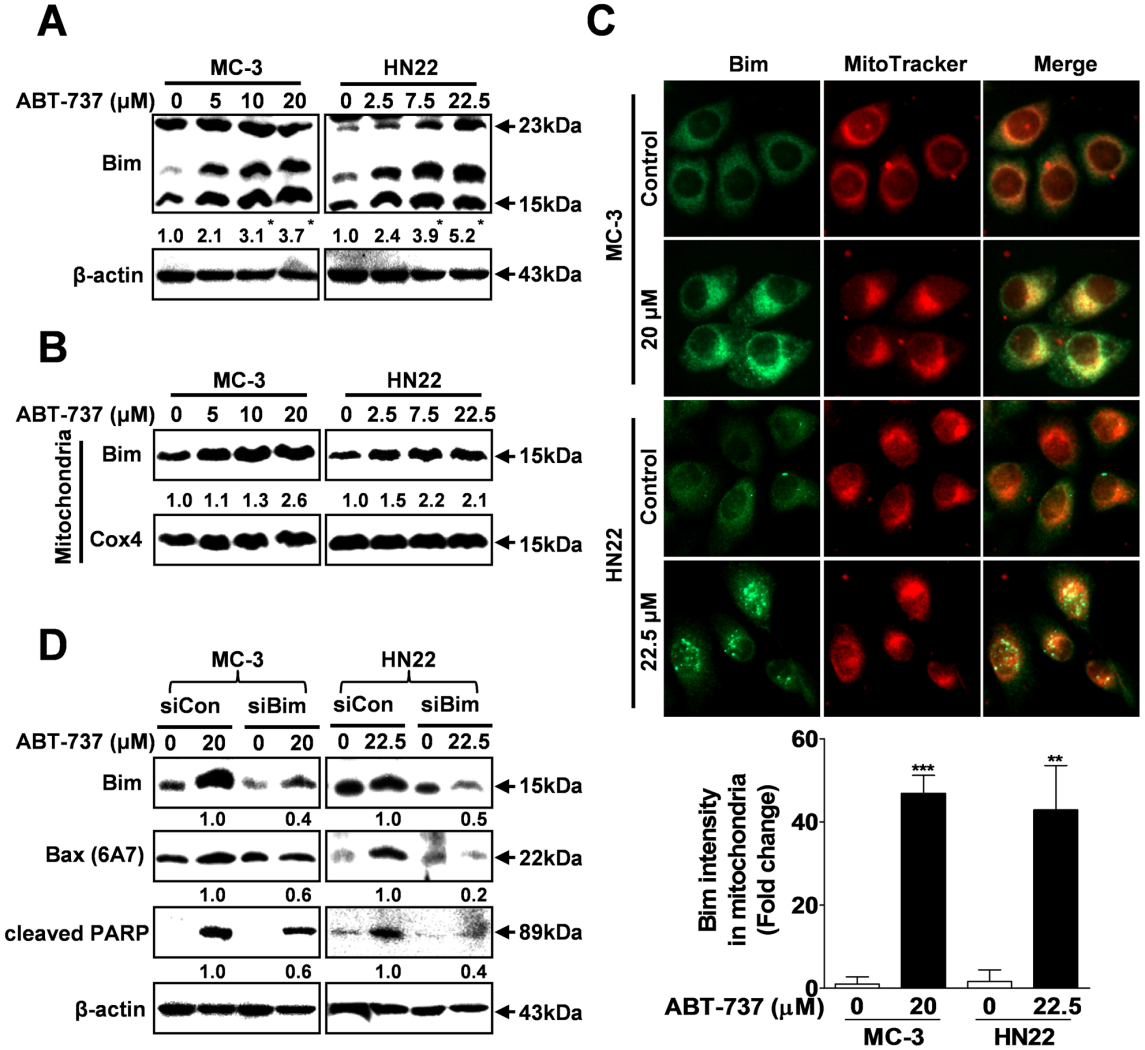
ABT-737 induces Bax-dependent apoptotic cell death via Bim activation MC-3 and HN22 cells were treated with or without ABT-737 for 24 hr, total cellular protein was prepared, and the protein levels of total Bim were evaluated by Western blot analysis **A.** MC-3 and HN22 cells were treated with or without ABT-737 for 24 hr; afterwards, mitochondrial fractions were prepared, and the protein levels of total Bim were evaluated by Western blot analysis **B.** Immunocytochemistry revealed the mitochondrial translocation of Bim from the cytoplasm after ABT-737 treatment (20 μM in MC-3 cells and 22.5 μM in HN22 cells) for 24 hr. MitoTracker (red) staining labels mitochondria within each field **C.** MC-3 and HN22 cells were transfected with either control siRNA (siCon) or siRNA specific to Bim (siBim); afterwards, cells were treated with or without ABT-737. Western blot analysis was performed to evaluate the expression levels of Bim, active Bim (6A7) and cleaved-PARP **D.** β-actin was used as an internal control. The Cox4 exclusive mitochondria marker was used as a control for separating mitochondrial and cytosolic fractions. The results are shown as the mean ± SD from three independent experiments. *P < 0.05, **P < 0.01, and ***P < 0.001.

We investigated the effects of ABT-737 treatment on the basal expression levels of Bcl-2 family proteins using Western blot analysis. Bim expression is associated with ABT-737 treatment (Figure 4A); however, we did not demonstrate any link between anti-apoptotic family members (Bcl-2 and Bcl-xL) and ABT-737 treatment (Supplementary Figure S1). Bim is normally sequestered to the microtubule-associated dynein motor complex in the cytoplasm. Following certain apoptotic stimuli, Bim is translocated to the mitochondria, where it causes cytochrome c release and apoptosis [25]. Western blot analysis revealed mitochondrial translocation of Bim from the cytoplasm after ABT-737 treatment (Figure 4B). Consistent with the Western blot analysis results, Bim (green fluorescence) was mainly localized in the mitochondria of cells treated with ABT-737, as shown by co-localization with the red–fluorescent MitoTracker (Figure 4C). To more specifically address the direct link between Bim and ABT-737-mediated apoptosis, MC-3 and HN22 cells were pre-transfected with Bim-specific siRNA before ABT-737 treatment. Bim knockdown successfully attenuated the ABT-737-induced activation of the pro-apoptotic molecules Bax and cleaved PARP (Figure 4D). Therefore, Bim may act as an upstream modulator of Bax-mediated apoptotic cell death.

### ABT-737 regulates bim expression through transcriptional or post-translational mechanisms

**Figure 5:**
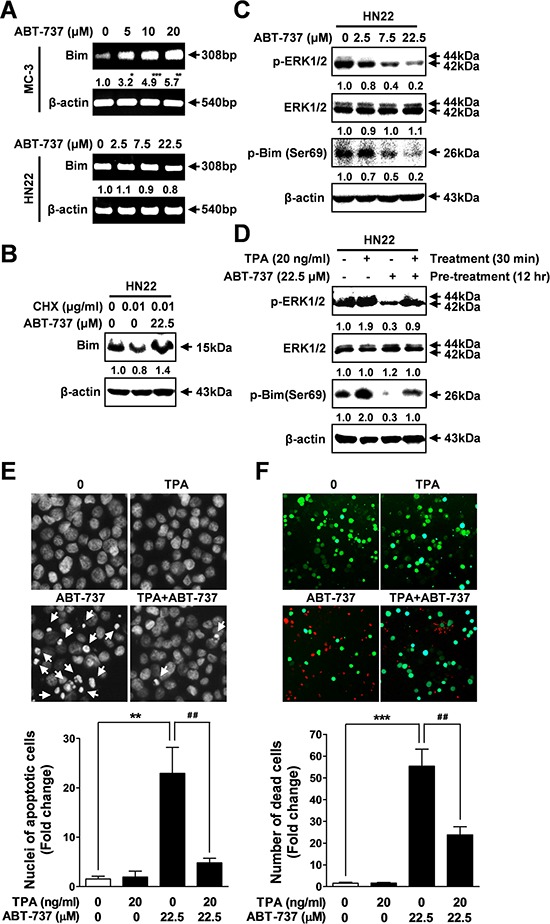
ABT-737 regulates Bim expression through post-translational modifications via the ERK1/2 signaling pathway MC-3 and HN22 cells were treated with or without ABT-737, and the mRNA levels of Bim were analyzed by RT-PCR **A.** HN22 cells were preincubated with a protein synthesis inhibitor CHX (0.01 μg/ml) for 1 hr prior to treatment with ABT-737 for 24 hr; afterwards, cells were harvested for Western blot analysis to detect Bim expression **B.** The expression levels of p-ERK1/2, total ERK1/2, and p-Bim (Ser69) were analyzed by Western blot **C.** HN22 cells were preincubated with a TPA (a phorbol ester, 20 ng/ml) for 30 min prior to treatment with ABT-737 for 12 h; afterwards, cells were harvested for Western blot analysis to detect p-ERK1/2, total ERK1/2, and p-Bim (Ser69) **D.** HN22 cells were co-incubated with a TPA (20 ng/ml) and ABT-737 for 12 hr;, after which nuclear fragmentation and condensation were evaluated by DAPI staining with fluorescence microscopy (magnification X400) **E.** Qualitative estimation of ABT-737-induced apoptotic cell death was obtained by a live/dead assay, which differentially labels live (green) and dead (red) cells with fluorescent dyes **F.** β-actin was used as an internal control. The results are shown as the mean ± SD from three independent experiments. *P < 0.05, **P < 0.01, and ***P < 0.001.

Previous studies suggest that ABT-737 inhibits the growth of human cancers by increasing Bim expression [26, 27], but it is unclear how Bim expression is regulated by ABT-737 treatment. Therefore, we examined whether ABT-737 treatment could affect the mRNA expression level of Bim in both cell lines. Interestingly, the mRNA levels of Bim were significantly increased in response to ABT-737 treatment in a concentration dependent manner in MC-3 cells; however, the Bim mRNA was not affected by ABT-737 treatment in HN22 cells (Figure 5A). Next, to determine whether ABT-737 had an effect on the initiation of Bim protein synthesis, HN22 cells were preincubated with the protein synthesis inhibitor cycloheximide (CHX) before ABT-737 exposure. CHX did not rescue Bim from the ABT-737-induced up-regulation of protein levels (Figure 5B), indicating that ABT-737 induces Bim up-regulation in a translation-independent manner in HN22 cells.

### ABT-737 regulates bim expression through post-translational modifications by controlling the ERK1/2 signaling pathway

Bim expression can be negatively regulated at the post-translational level by controlling ERK1/2 phosphorylation [28]. Recently, it has been demonstrated that Bim phosphorylation via ERK1/2 on serine 69 promotes its ubiquitination and subsequent proteasomal degradation [29]. Next, we examined whether ABT-737 could regulate the ERK1/2 signaling pathway in HN22 cells. ABT-737 treatment markedly suppressed the ERK1/2 phosphorylation levels in a concentration-dependent manner (Figure 5C). Interestingly, the suppression of ERK1/2 phosphorylation levels was tightly associated with decreased phosphorylation levels of Bim on serine 69 (Figure 5C). Next, we applied a potent activator of ERK/12 signaling to further demonstrate its regulatory role in ABT-737-mediated Bim expression. Activation of ERK1/2 signaling with TPA, a known MAPK activator, successfully attenuated the ABT-737-induced Bim dephosphorylation compared to controls (Figure 5D). Consistently, we have shown that activation of ERK1/2 signaling by TPA treatment successfully attenuates ABT-737-induced DNA fragmentation (Figure 5E) as well as the -live/dead ratio (Figure 5F), indicating that ERK1/2 signaling may play the primary regulatory role in the ABT-737-induced pro-apoptotic Bim signaling pathway, acting as its upstream regulator.

### Therapeutic effects of ABT-737 in a xenograft model of human oral cancer

**Figure 6:**
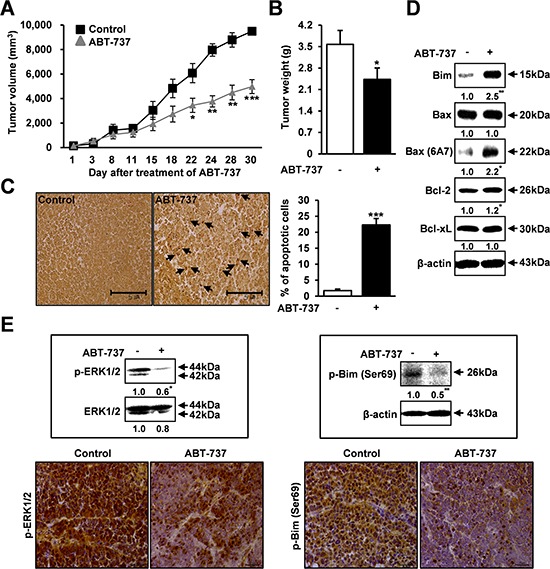
Therapeutic effects of ABT-737 in a nude mice xenograft model of human oral cancer The mice were implanted with MC-3 cells with s.c. injection into the flanks. Mice were treated with 50 mg/kg/day ABT-737 (i.p.) five times per week for 30 days. The tumor volume was measured two times per week. ABT-737 treatment significantly reduced the average tumor volume and weight compared to PBS controls **A, B.** ABT-737 treatment resulted in increased TUNEL-positive cells in representative sections from tumor xenografts **C.** Protein lysates were prepared from tumor xenografts, and the protein levels of Bim, total Bax, active Bax (6A7), Bcl-2, and Bcl-xL were evaluated by Western blot analysis **D.** The p-ERK1/2 expression levels were analyzed by Western blot analysis and immunohistochemistry in tumor xenografts **E.** β-actin was used as an internal control. The results are shown as the mean ± SD from three independent experiments. *P < 0.05, **P < 0.01, and ***P < 0.001.

**Figure 7:**
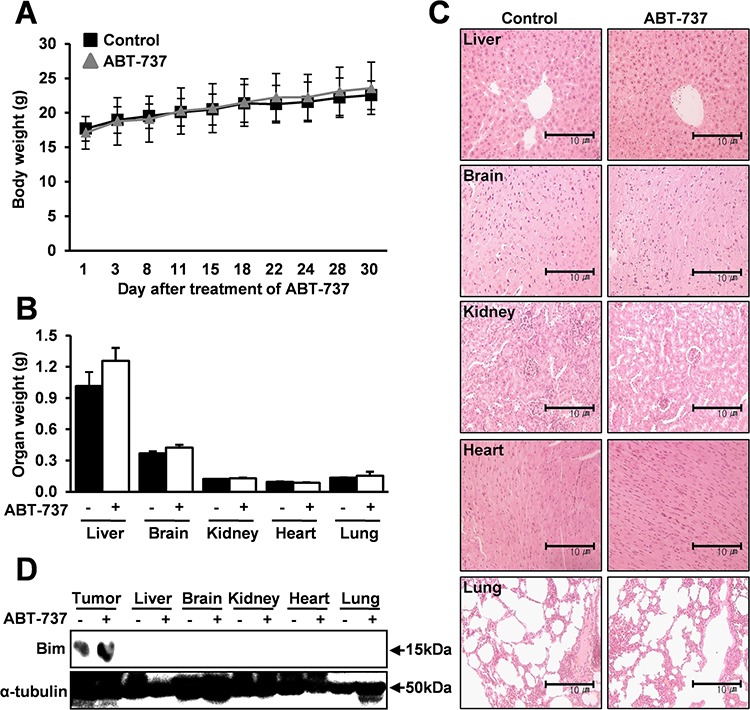
No marked signs of systemic toxicity with the ABT-737 treatment The mice were implanted with MC-3 cells with s.c. injection into the flanks. Mice were treated with 50 mg/kg/day ABT-737 (i.p.) five times per week for 30 days. The body weight was measured two times per week. No significant mouse body and organ weight loss was observed in the ABT-737-treated groups **A, B.** The microscopic pathology of major organs shows no evidence of adverse systemic toxicity up to 50 mg/kg/day ABT-737 treatment in the mice **C.** High levels of Bim expression were observed in only tumors, whereas its expression is substantially undetectable in other organs **D.** α-tubulin was used as an internal control. The results are shown as the mean ± SD from three independent experiments. *P < 0.05, **P < 0.01, and ***P < 0.001.

Based on these *in vitro* results, we further investigated the *in vivo* antitumor effect of ABT-737 using a mouse xenograft model. ABT-737 (50 mg/kg) was administered by systemic injections that were given five times per week, and the tumor size was measured for 30 days. Importantly, there was a significant and consistent reduction in the tumor volume and weight in the mice that were treated with ABT-737 compared to the control groups (Figure 6A and 6B). *In vivo* xenograft results also demonstrated that ABT-737 treatment resulted in increased TUNEL-positive cells in tumor xenografts (Figure 6C). Furthermore, to determine whether and to what extent ABT-737 treatment affects the expressions of Bcl-2 family proteins *in vivo*, we also compared the relative expression levels of Bcl-2 proteins, such as Bax, Bim, Bcl-2, and Bcl-xL, in tumors with or without ABT-737 treatment. Consistent with our *in vitro* results, the relative expression levels of Bax and Bim were significantly increased in the ABT-737 treated groups compared with those in the control groups (Figure 6D). Moreover, ABT-737 treatment significantly suppressed the phosphorylation levels of ERK1/2 and Bim in tumor xenografts (Figure 6E). No significant mouse body (Figure 7A) or organ (Figure 7B) weight loss was observed in the ABT-737-treated groups, indicating that the ABT-737-associated toxicity was minimal. No notable intergroup differences were observed among the organs, indicating that there were no marked signs of systemic toxicity at the ABT-737 dose used in this study (Figure 7C). High levels of Bim expression were observed in only tumors, whereas the expression was substantially undetectable in other organs, including the liver, brain, kidney, heart, and lung (Figure 7D), supporting the highly specific *in vivo* on-target effects of ABT-737.

## DISCUSSION

Bcl-2 family proteins modulate the mitochondrial out membrane potential (MOMP) by their specific communications and finally cells are going to either live or die. Thus, targeting Bcl-2 family appeared as a clinically attractive apoptosis-based strategy for chemotherapy. ABT-737 was discovered by the fragment-screening approach based on structure/activity relationship by nuclear magnetic resonance [30]. It was extensively characterized in preclinical studies and the therapeutic activity of ABT-263 (navitoclax), its oral bioavailable derivative in patients with hematologic malignancies [31–34] meaning that many efforts are necessary to do the clinical application of these kinds of BH3 mimetics. An important but challenging task in the development of new chemotherapeutic drug, such as a BH3 mimetic, is to determine its precise biological targets and mechanisms of action. We hypothesized that any therapeutic drugs mimicking the BH3-only proteins might act through their identified downstream target, Bax [35–37]. Therefore, we examined the selective ability to regulate Bax expression in human oral squamous cell carcinoma HN22 cells and salivary gland mucoepidermoid carcinoma MC-3 cells. In this study we found that the anticancer effect of ABT-737 on human oral cancer cells seems to be mediated through the BH3 domain protein, Bim, which directly activates Bax to trigger apoptosis rather than regulating other Bcl-2 family proteins, such as Bcl-2 and Bcl-xL.

Bim expression can be regulated by a dual-component mechanism involving transcriptional and post-translational regulation resulting in the antitumor activities of ABT-737 against human oral cancer cells *in vitro* and *in vivo*. Especially, the best-characterized post-translational modification that regulates Bim expression is phosphorylation on serine 69, which promotes the proteasome-dependent degradation of Bim. Previously, it was shown that activation of the ERK1/2 signaling pathway promotes phosphorylation at serine 69 and consequent proteasome-dependent degradation of Bim [38–40]. Consistently, following treatment with ABT-737, we found that Bim expression is post-translationally upregulated in HN22 cells through ERK1/2 signaling, allowing them to gain their full apoptotic function. Phosphorylation at serine 69 via the ERK1/2 signaling pathway seems to be a critical transduction step for Bim turnover because serine 69 mutants are defective for proteasome-dependent degradation, accumulate at higher levels in cells and exhibit enhanced cell death by apoptosis [29, 41]. Interestingly, in addition to promoting the post-translational turnover of Bim in HN22 cells, the pro-apoptotic effect of ABT-737 seems to be mediated through transcriptionally increased Bim expression levels in MC-3 cells. Consistent with previous results [42–44], our findings suggest that ABT-737-mediated Bim expression may be in a cell type-specific manner (Figure 5).

An ideal therapeutic agent would specifically target the cancer cells without causing overwhelming systemic damage. However, most conventional chemotherapeutic agents are associated with serious cytotoxicity, which is largely due to the presence of their targets in normal healthy cells. Specific actions with high efficacy are needed for a good drug candidate because high selectivity for its biological targets would be nontoxic to the patient through limiting undesirable cytotoxicity. No significant mouse body/organ weight loss (Figure 7A and 7B) and no notable intergroup differences among organs (Figure 7C) were observed in the ABT-737-treated groups, indicating that there were no marked signs of systemic toxicity at the dose used in this study. Importantly, high Bim levels were observed in only tumors, whereas Bim expression was virtually undetectable in other organs (Figure 7D), supporting the highly specific *in vivo* on-target effects of ABT-737. However, as ABT-737 targets the Bim/Bax pro-apoptotic signaling pathway, it could be expected to cause cytotoxic effects *in vivo* that are associated with some of the developmental abnormalities in mice lacking these pro-apoptotic Bcl-2 proteins [45, 46]. Therefore, more detailed *in vivo* studies are needed to generate more direct evidence for the undesirable side effects of ABT-737.

Interestingly, we also found that Bcl-xL and Bcl-2 are not targeted by ABT-737 in either human oral cancer cell line (Supplementary Figure S1). Therefore, it would be reasonable to postulate that Bcl-xL and Bcl-2 contribute to ABT-737 resistance in human oral cancer. Mérino et al. [47] reported that increasing Bcl-xL expression conferred resistance to ABT-737, although Bim expression was also increased, in leukemic cells, whereas Delft et al. [34] found that Bcl-xL- overexpressing embryonic fibroblasts were significantly less sensitive to ABT-737-induced apoptosis than Bcl-2-overexpressing cells. Furthermore, high expression of Bcl-2 but not Bcl-xL correlates with sensitivity to ABT-737 in chronic lymphocytic leukemia [13, 48]. Additionally, Tahir et al. reported that high levels of Bcl-xL correlate with sensitivity to ABT-263 (an orally available analog of ABT-737) in lung carcinoma [49]. Therefore, it seems possible that Bcl-xL and Bcl-2 can mediate resistance to ABT-737 in some cancer cell types but that they are specifically targeted in others. More detailed studies will be needed to dissect the roles of ABT-737 resistance in human oral cancer.

**Figure 8:**
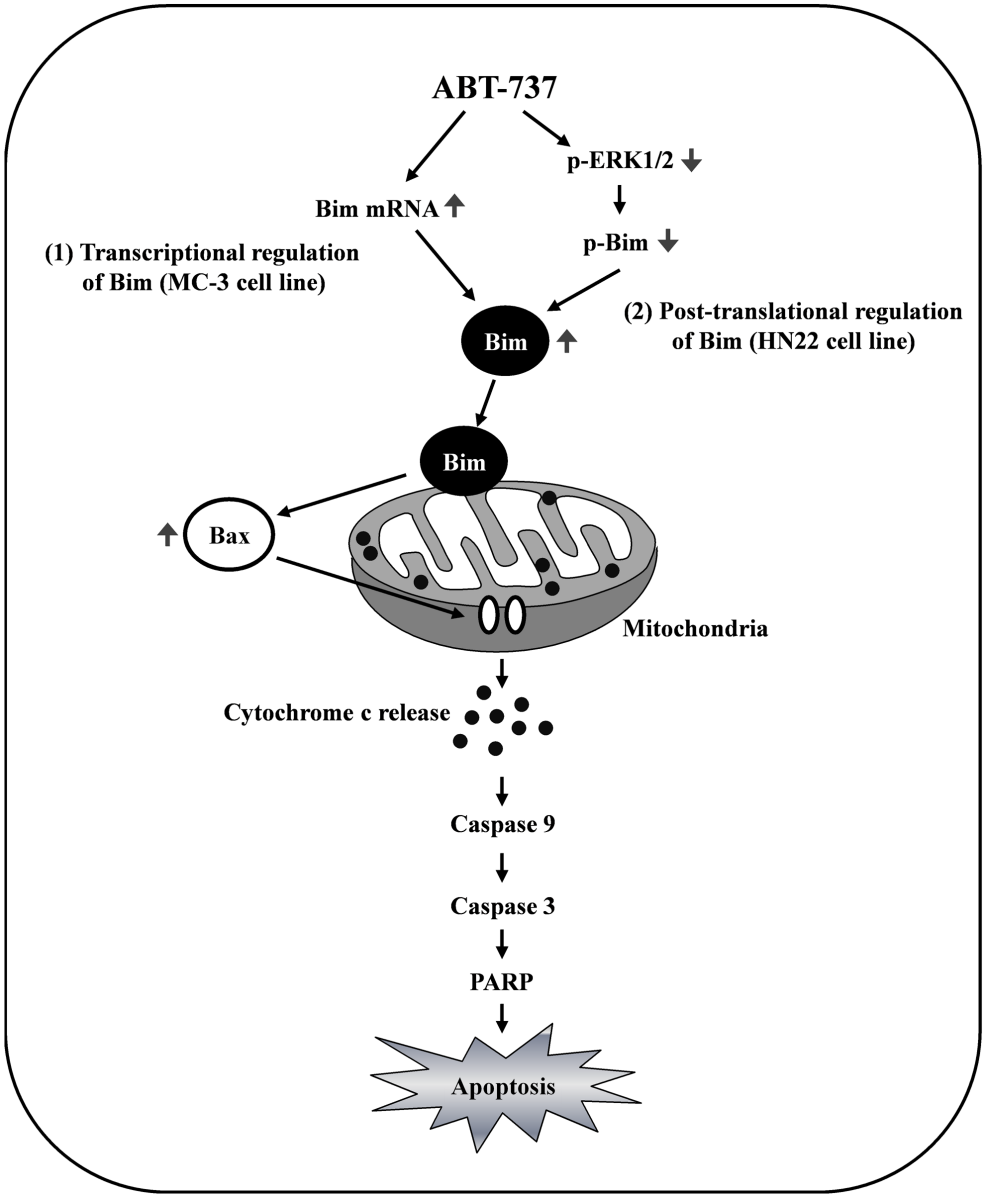
Schematic summary of the effects of ABT-737 on human oral cancer cells The Bim expression levels were significantly increased in response to ABT-737 treatment in MC-3 human mucoepidermoid carcinoma cells, whereas Bim phosphorylation by ERK1/2 on serine 69 promotes its ubiquitination and subsequent proteasomal degradation in HN22 human oral squamous carcinoma cells. The inactivation of the ERK1/2 signaling pathway by ABT-737 treatment reduces the Bim expression levels via both transcriptional and posttranslational regulation in a cell type-dependent manner, inducing mitochondria-mediated apoptosis of human oral cancer cells.

The MAPK signaling pathway has received considerable attention in recent years for the discovery and development of cancer-related therapeutic agents due to its potential to promote the proliferation and survival of various cancer cell types [50]. Among each element in the MAPK signaling cascades, ERK1/2 is considered an attractive target for developing therapeutics against cancers due to its significant pharmacological specificity and selectivity [51]. Although the precise mechanisms of how BH3 mimetics promote Bcl-2 family-mediated apoptosis in human oral cancer cells are still largely unknown, previous studies have established the role of the ERK1/2 signaling pathway in regulating cancer cell growth and apoptosis [52]. Recently, a link between the ERK1/2 signaling pathway and Bim has been suggested [53, 54]. Based on more recent evidence, the inhibition of certain oncogenic proteins with specific inhibitors inactivates the ERK1/2 signaling cascade and increases Bim-mediated apoptotic cell death [55]. These agents do not only directly target Bim transcription itself they also suppress ERK1/2 signaling indirectly, causing the inactivation of key signaling pathways that reduce the Bim protein levels [54]. Therefore, it is possible that the antitumor effects of ABT-737 on the oral cancer cells may contribute to Bim regulation both at the transcriptional and/or post-translational levels via suppressing the ERK1/2 signaling pathway. In agreement with our hypothesis, ABT-737 exposure markedly suppressed the phosphorylated ERK1/2 levels in HN22 human oral squamous cancer cells (Figure 5C). Consistently, activation of the ERK1/2 signaling pathway with a potent MAPK activator TPA was successfully attenuated by ABT-737-induced Bim dephosphorylation (Figure 5D) and apoptosis (Figure 5E and 5F) compared to the controls. As a result, we conclude that inactivation of the ERK1/2 signaling pathway by ABT-737 treatment reduces the Bim expression levels via both transcriptional and/or posttranslational regulation in a cell type-dependent manner, inducing the mitochondria-mediated apoptosis of human oral cancer cells (Figure 8). To the best of our knowledge, this is the first demonstration of the anti-cancer effects of ABT-737 on human oral cancer.

## MATERIALS AND METHODS

### Cell culture and chemical treatment

MC-3 (human mucoepidermoid carcinoma) cells were provided from Prof. Wu Junzheng (Forth Military Medical University, Xi'an, China) and HN22 (human oral squamous carcinoma) cells were obtained from Prof. Chong Heon Lee (Dankook University, Cheonan, Korea). MC-3 and HN22 cells were cultured in DMEM supplemented with 10% FBS and antibiotics. Each cell lines were maintained at 37°C in 5% CO_2_ atmosphere. All experiments were prepared in cells cultured at 50% ~ 60% confluence. ABT-737 (Selleck Chemicals, Houston, TX, USA) was dissolved in DMSO, aliquoted, and stored at −20°C. Final concentration of DMSO did not exceed 0.1%.

### 3-(4,5-dimethylthiazol-2-yl)-5-(3-carboxymethoxyphenyl)-2-(4-sulfophenyl)-2H-tetrazolium (MTS) assay

Cell viability was determined by MTS assay (Promega, Madison, WI, USA). Cells were seeded in 96-well plates and incubated with ABT-737 for 24 hr. Briefly, MTS solution was added to each well and maintained for 1 hr at 37°C. The absorbance was measured at 482 nm using a Chameleon microplate reader (Hidex, Turku, Finland).

### Trypan blue exclusion assay

The growth inhibitory effect of ABT-737 was determined with trypan blue solution (Gibco, Paisley, UK). Detached cells were stained with trypan blue (0.4%), and then viable cells were counted using a hemocytometer.

### Live/dead assay

The cytotoxicity of ABT-737 was determined using Live/Dead & Viability/Cytotoxicity assay (Life Technologies, Grand Island, NY, USA). The polyanionic dye Calcein-AM is retained within live cells, producing an intense green fluorescence through intracellular esterase activity. Ethidium homodimer-1 enters cells with damaged membranes and binds to nucleic acids, producing a bright red fluorescence in dead cells. Briefly, cells were stained with 2 μM Calcein-AM and 4 μM Ethidium homodimer-1, and then incubated for 30 min at RT. Cells were analyzed under a fluorescence microscopy (Carl Zeiss, Oberkochen, Germany) with the appropriate excitation and emission filters.

### 4′-6-diamidino-2-phenylindole (DAPI) staining

To detect of nuclear morphological changes of apoptotic cells, cells were stained with DAPI solution (Sigma-Aldrich, Louis, MO, USA). Briefly, detached cells were fixed in 100% methanol at RT for 10 min, deposited on slides, and stained with DAPI solution (2 μg/ml). The morphological changes of apoptotic cells were observed under a fluorescence microscopy.

### Western blot analysis

Whole-cell lysates were prepared with lysis buffer and protein concentration in each sample was measured using a DC Protein Assay Kit (BIO-RAD Laboratories, Madison, WI, USA). After normalization, equal amounts of protein were separated by SDS-PAGE and then transferred to Immun-Blot™ PVDF membranes. The membranes were blocked with 5% skim milk in TBST at RT for 2 hr, and incubated with primary antibodies and corresponding HRP-conjugated secondary antibodies. Antibodies against cleaved PARP, cleaved caspse-3, cleaved caspase-9, Bax, Bcl-2, Bcl-xL, p-ERK1/2 (Thr202/Tyr204), ERK1/2, p-Bim (Ser69), Bim, Bad and Bid were purchased from Cell Signaling Technology, Inc., (Charlottesville, VA, USA). Actin and α-tubulin antibodies were obtained from Santa Cruz Biotechnology, Inc., (Santa Cruz, CA, USA). Bax (6A7) antibody was from BD Pharmingen™ (San Jose, CA, USA) and Cox4 antibody was from Abcam (Cambridge, UK). The immunoreactive bands were visualized by ImageQuant™ LAS 500 (GE Healthcare Life Sciences, Piscataway, NJ, USA).

### Mitotracker staining

Cells were seeded in 4-well plates, and then treated with DMSO or ABT-737 for 24 hr. To label mitochondria, cells were incubated with 500 nM MitoTracker^®^ Mitochondrion-Selective Probes (Invitrogen, Carlsbad, CA, USA) at 37°C for 45 min, which passively diffuse across the plasma membrane and accumulate in active mitochondria. After staining live cells with a MitoTracker probes, cells were fixed and permeabilized using a Cytofix/Cytoperm Solution at 4°C for 1 hr. Cells were blocked with 1% BSA at RT for 1 hr and incubated at 4°C overnight, followed by incubation with FITC-conjugated secondary antibody at RT for 1 hr. Cells were observed by fluorescence microscopy with the appropriate filters for fluorescence dye.

### Detection of bax conformational change

Whole-cell lysates were extracted by lysis buffer, and then proteins were analyzed by Western blot. For detection of Bax conformational change, proteins were detected using specific primary antibody for the active form of Bax (6A7).

### Preparation of cytosolic and mitochondrial fractions

To determine the effect of ABT-737 on Bax translocation into mitochondria, cytosolic extracts were prepared by plasma membrane extraction buffer containing 0.05% digitonin at RT for 1 hr. The lysates were centrifuged at 13,000 rpm at 4°C for 5 min to separate supernatant containing cytosolic proteins. Pellets were washed and resuspended by plasma membrane extraction buffer containing 0.5% Triton X-100 at ice for 10 min, and then centrifuged 13,000 rpm at 4°C for 5 min. The supernatant from this last centrifugation contained the mitochondrial proteins.

### Crosslinking

To detect intracellular Bax oligomerization, cells were suspended by conjugation buffer containing 10 mM EDTA. The lysates were maintained with 2 mM Bismaleimide Crosslinker (Thermo Scientific, Rockford, IL, USA) for conjugation between sulfhydryl groups at RT for 1 hr, and then extracted by lysis buffer for Western blot analysis.

### Small interfering RNA (siRNA)

A control nonspecific siRNA and Bim-targeted siRNA were obtained from Santa Cruz Biotechnology (Santa Cruz, CA, USA). Cells were seeded in 6-well plates and transfected at 50% confluency with 50 nM siRNA using siRNA transfection reagent (Santa Cruz, CA, USA). After transfection for 6 hr, cells were treated with DMSO or ABT-737 for 24 hr.

### Reverse transcription-polymerase chain reaction (RT-PCR)

Total RNA was extracted by easy-BLUE Total RNA Extraction Kit (INTRON, Daejeon, Korea) according to the manufacturer's instructions. One microgram of RNA was reverse transcribed by TOPscript™ RT DryMIX (Elpis Biotech, Daejeon, Korea), and the resultant cDNA was subjected to PCR using HiPi PCR PreMix (Elpis Biotech, Daejeon, Korea). The PCR condition of Bim was as follows: (30 cycles: 1 min at 94°C, 1 min at 62°C and 1 min at 72°C), and the PCR condition of β-actin was as follows: (28 cycles: 1 min at 94°C, 1 min at 60°C and 1 min at 72°C). The primer sequences were used: Bim sense 5′-ATG GCA AG CAA CCT TCT GA-3′, Bim anti-sense 5′-CTG TCT GTG TCA AAA GAG-3′, β-actin sense 5′-GTG GGG CGC CCC AGG CAC CA-3′, β-actin anti-sense 5′-CTC CTT AAT GTC ACG CAC GAT TTC-3′. The intensities of each band were normalized to β-actin. The amplified products were analyzed by 2% agarose gel electrophoresis and stained with ethidium bromide.

### Nude mouse xenograft assay

Female nude mice were purchased from Orient Ltd (Suwon, Korea). All mice were handled according to the Institutional Animal Care and Use Committee (IACUC) guidelines. MC-3 cells were inoculated with s.c. injection into the flanks of the mice, and then mice were randomly assigned to 2 treatment groups (*n* = 4 for each group). The treatment groups were received 50 mg/kg/day of ABT-737 (i.p.) five times per week for 30 days. Tumor volume and body weight were measured two times per week. After 30 days, tumor weight and organ weight were measured. The tumors were measured along the two diameter axis with calibers to allow a calculation of tumor volume using the following formula: V = π/6 {(D + d)/2}^3^, where D and d were the larger and smaller diameters, respectively.

### Terminal deoxynucleotidyl transferase dUTP nick end labeling (TUNEL) assay

Paraffin-embedded tumor tissues were analyzed by TUNEL *in situ* apoptosis detection kit (Dead-End Colorimetric TUNEL system, Promega, Madison, WI, USA) according to the manufacturer's instructions. Briefly, Paraffin-embedded sections were deparaffinized and rehydrated. The sections were incubated with proteinase K for 15 min at RT and then the endogenous peroxidase was block with 0.3% hydrogen peroxide for 5 min. The digoxigenine-dUTP end labeled DNA was detected using an anti-digoxigenin peroxidase antibody followed by peroxidase detection with 0.05% DAB containing 0.02% hydrogen peroxide. The sections were counterstained with methyl green, and then the brown-colored apoptotic bodies in the tumor sections from control and ABT-737-treated mice were counted using Nikon Eclipse E800 microscope (Nikon Inc., Melville, NY, USA). Randomly five selected fields were visualized under a light microscope at a 400 magnification and the number of positively stained cells was counted.

### Immunohistochemistry

Unstained tissue sections were deparaffinized, treated with 100% alcohol, and washed with PBS. For antigen retrieval, sections were boiled in citric buffer (pH 6.0) for 10 min using a hot plate and then cooled for 1 hr at RT. Endogenous peroxidase was blocked with peroxidase blocking solution (Sigma-Aldrich) for 10 min, and the sections were treated with protein blocking solution for 20 min. p-ERK1/2 (Thr202/Tyr204) and p-Bim (Ser69) antibodies (Cell Signaling Technology, Inc.) were applied overnight at 4°C and sections were incubated using secondary antibodies 1 hr at 37°C. Sections were then stained with freshly prepared DAB substrate (Dako, Glostrup, Denmark), counterstained with Mayer's hematoxylin, dehydrated, and mounted and examined under a light microscope.

### Histopathological examination of organs

Mice organs including liver, brain, kidney, heart and lung were fixed in 10% neutral buffered formalin. Tissue sections were cut at a thickness of 4 μm and stained with hematoxylin and eosin (H&E). Histopathological changes were analyzed using Nikon Eclipse E800 microscope.

### Statistical analysis

Student's *t*-test was used to determine the significance of differences between the control and treatment groups; *p* values of < 0.05 were considered significant.

## SUPPLEMENTARY FIGURE



## References

[1] Hanahan D, Weinberg RA (2011). Hallmarks of cancer: the next generation. Cell.

[2] Reed JC, Pellecchia M (2005). Apoptosis-based therapies for hematologic malignancies. Blood.

[3] Fulda S, Galluzzi L, Kroemer G (2010). Targeting mitochondria for cancer therapy. Nature reviews Drug discovery.

[4] Chipuk JE, Moldoveanu T, Llambi F, Parsons MJ, Green DR (2010). The BCL-2 family reunion. Mol Cell.

[5] Youle RJ, Strasser A (2008). The BCL-2 protein family: opposing activities that mediate cell death. Nat Rev Mol Cell Biol.

[6] Simoes-Wust AP, Schurpf T, Hall J, Stahel RA, Zangemeister-Wittke U (2002). Bcl-2/bcl-xL bispecific antisense treatment sensitizes breast carcinoma cells to doxorubicin, paclitaxel and cyclophosphamide. Breast Cancer Res Treat.

[7] Brotin E, Meryet-Figuiere M, Simonin K, Duval RE, Villedieu M, Leroy-Dudal J, Saison-Behmoaras E, Gauduchon P, Denoyelle C, Poulain L (2010). Bcl-XL and MCL-1 constitute pertinent targets in ovarian carcinoma and their concomitant inhibition is sufficient to induce apoptosis. Int J Cancer.

[8] Baell JB, Huang DC (2002). Prospects for targeting the Bcl-2 family of proteins to develop novel cytotoxic drugs. Biochem Pharmacol.

[9] Fesik SW (2005). Promoting apoptosis as a strategy for cancer drug discovery. Nat Rev Cancer.

[10] Rutledge SE, Chin JW, Schepartz A (2002). A view to a kill: ligands for Bcl-2 family proteins. Curr Opin Chem Biol.

[11] Certo M, Del Gaizo Moore V, Nishino M, Wei G, Korsmeyer S, Armstrong SA, Letai A (2006). Mitochondria primed by death signals determine cellular addiction to antiapoptotic BCL-2 family members. Cancer cell.

[12] Oltersdorf T, Elmore SW, Shoemaker AR, Armstrong RC, Augeri DJ, Belli BA, Bruncko M, Deckwerth TL, Dinges J, Hajduk PJ, Joseph MK, Kitada S, Korsmeyer SJ, Kunzer AR, Letai A, Li C (2005). An inhibitor of Bcl-2 family proteins induces regression of solid tumours. Nature.

[13] Del Gaizo Moore V, Brown JR, Certo M, Love TM, Novina CD, Letai A (2007). Chronic lymphocytic leukemia requires BCL2 to sequester prodeath BIM, explaining sensitivity to BCL2 antagonist ABT-737. J Clin Invest.

[14] Tagscherer KE, Fassl A, Campos B, Farhadi M, Kraemer A, Bock BC, Macher-Goeppinger S, Radlwimmer B, Wiestler OD, Herold-Mende C, Roth W (2008). Apoptosis-based treatment of glioblastomas with ABT-737, a novel small molecule inhibitor of Bcl-2 family proteins. Oncogene.

[15] Tahir SK, Yang X, Anderson MG, Morgan-Lappe SE, Sarthy AV, Chen J, Warner RB, Ng SC, Fesik SW, Elmore SW, Rosenberg SH, Tse C (2007). Influence of Bcl-2 family members on the cellular response of small-cell lung cancer cell lines to ABT-737. Cancer Res.

[16] Vattemi E, Graiff C, Sava T, Pedersini R, Caldara A, Mandara M (2008). Systemic therapies for recurrent and/or metastatic salivary gland cancers. Expert Rev Anticancer Ther.

[17] Agoni L, Basu I, Gupta S, Alfieri A, Gambino A, Goldberg GL, Reddy EP, Guha C (2014). Rigosertib is a more effective radiosensitizer than cisplatin in concurrent chemoradiation treatment of cervical carcinoma, *in vitro* and *in vivo*. Int J Radiat Oncol Biol Phys.

[18] Scripture CD, Figg WD, Sparreboom A (2006). Peripheral neuropathy induced by paclitaxel: recent insights and future perspectives. Curr Neuropharmacol.

[19] Li P, Nijhawan D, Budihardjo I, Srinivasula SM, Ahmad M, Alnemri ES, Wang X (1997). Cytochrome c and dATP-dependent formation of Apaf-1/caspase-9 complex initiates an apoptotic protease cascade. Cell.

[20] Eskes R, Desagher S, Antonsson B, Martinou JC (2000). Bid induces the oligomerization and insertion of Bax into the outer mitochondrial membrane. Mol Cell Biol.

[21] Gross A, Jockel J, Wei MC, Korsmeyer SJ (1998). Enforced dimerization of BAX results in its translocation, mitochondrial dysfunction and apoptosis. EMBO J.

[22] Wolter KG, Hsu YT, Smith CL, Nechushtan A, Xi XG, Youle RJ (1997). Movement of Bax from the cytosol to mitochondria during apoptosis. J Cell Biol.

[23] Wei MC, Zong WX, Cheng EH, Lindsten T, Panoutsakopoulou V, Ross AJ, Roth KA, MacGregor GR, Thompson CB, Korsmeyer SJ (2001). Proapoptotic BAX and BAK: a requisite gateway to mitochondrial dysfunction and death. Science.

[24] Antonsson B, Montessuit S, Sanchez B, Martinou JC (2001). Bax is present as a high molecular weight oligomer/complex in the mitochondrial membrane of apoptotic cells. J Biol Chem.

[25] Puthalakath H, Huang DC, O'Reilly LA, King SM, Strasser A (1999). The proapoptotic activity of the Bcl-2 family member Bim is regulated by interaction with the dynein motor complex. Mol Cell.

[26] Mazumder S, Choudhary GS, Al-Harbi S, Almasan A (2012). Mcl-1 Phosphorylation defines ABT-737 resistance that can be overcome by increased NOXA expression in leukemic B cells. Cancer Res.

[27] Wang H, Yang YB, Shen HM, Gu J, Li T, Li XM (2012). ABT-737 induces Bim expression via JNK signaling pathway and its effect on the radiation sensitivity of HeLa cells. PLoS One.

[28] Hughes R, Gilley J, Kristiansen M, Ham J (2011). The MEK-ERK pathway negatively regulates bim expression through the 3′ UTR in sympathetic neurons. BMC Neurosci.

[29] Luciano F, Jacquel A, Colosetti P, Herrant M, Cagnol S, Pages G, Auberger P (2003). Phosphorylation of Bim-EL by Erk1/2 on serine 69 promotes its degradation via the proteasome pathway and regulates its proapoptotic function. Oncogene.

[30] Shuker SB, Hajduk PJ, Meadows RP, Fesik SW (1996). Discovering high-affinity ligands for proteins: SAR by NMR. Science.

[31] Lessene G, Czabotar PE, Colman PM (2008). BCL-2 family antagonists for cancer therapy. Nature reviews Drug discovery.

[32] Anderson MA, Huang D, Roberts A (2014). Targeting BCL2 for the treatment of lymphoid malignancies. Seminars in hematology.

[33] Roy MJ, Vom A, Czabotar PE, Lessene G (2014). Cell death and the mitochondria: therapeutic targeting of the BCL-2 family-driven pathway. British journal of pharmacology.

[34] van Delft MF, Wei AH, Mason KD, Vandenberg CJ, Chen L, Czabotar PE, Willis SN, Scott CL, Day CL, Cory S, Adams JM, Roberts AW, Huang DC (2006). The BH3 mimetic ABT-737 targets selective Bcl-2 proteins and efficiently induces apoptosis via Bak/Bax if Mcl-1 is neutralized. Cancer cell.

[35] Cheng EH, Wei MC, Weiler S, Flavell RA, Mak TW, Lindsten T, Korsmeyer SJ (2001). BCL-2, BCL-X(L) sequester BH3 domain-only molecules preventing BAX- and BAK-mediated mitochondrial apoptosis. Mol Cell.

[36] Lindsten T, Ross AJ, King A, Zong WX, Rathmell JC, Shiels HA, Ulrich E, Waymire KG, Mahar P, Frauwirth K, Chen Y, Wei M, Eng VM, Adelman DM, Simon MC, Ma A (2000). The combined functions of proapoptotic Bcl-2 family members bak and bax are essential for normal development of multiple tissues. Mol Cell.

[37] Zong WX, Lindsten T, Ross AJ, MacGregor GR, Thompson CB (2001). BH3-only proteins that bind pro-survival Bcl-2 family members fail to induce apoptosis in the absence of Bax and Bak. Genes Dev.

[38] Lang-Rollin I, Vekrellis K, Wang Q, Rideout HJ, Stefanis L (2004). Application of proteasomal inhibitors to mouse sympathetic neurons activates the intrinsic apoptotic pathway. J Neurochem.

[39] Ley R, Balmanno K, Hadfield K, Weston C, Cook SJ (2003). Activation of the ERK1/2 signaling pathway promotes phosphorylation and proteasome-dependent degradation of the BH3-only protein, Bim. J Biol Chem.

[40] Mouhamad S, Besnault L, Auffredou MT, Leprince C, Bourgeade MF, Leca G, Vazquez A (2004). B cell receptor-mediated apoptosis of human lymphocytes is associated with a new regulatory pathway of Bim isoform expression. J Immunol.

[41] Ley R, Ewings KE, Hadfield K, Howes E, Balmanno K, Cook SJ (2004). Extracellular signal-regulated kinases 1/2 are serum-stimulated “Bim(EL) kinases” that bind to the BH3-only protein Bim(EL) causing its phosphorylation and turnover. J Biol Chem.

[42] Gogada R, Yadav N, Liu J, Tang S, Zhang D, Schneider A, Seshadri A, Sun L, Aldaz CM, Tang DG, Chandra D (2013). Bim, a proapoptotic protein, up-regulated via transcription factor E2F1-dependent mechanism, functions as a prosurvival molecule in cancer. J Biol Chem.

[43] Ley R, Ewings KE, Hadfield K, Cook SJ (2005). Regulatory phosphorylation of Bim: sorting out the ERK from the JNK. Cell Death Differ.

[44] Putcha GV, Le S, Frank S, Besirli CG, Clark K, Chu B, Alix S, Youle RJ, LaMarche A, Maroney AC, Johnson EM (2003). JNK-mediated BIM phosphorylation potentiates BAX-dependent apoptosis. Neuron.

[45] Hutcheson J, Scatizzi JC, Bickel E, Brown NJ, Bouillet P, Strasser A, Perlman H (2005). Combined loss of proapoptotic genes Bak or Bax with Bim synergizes to cause defects in hematopoiesis and in thymocyte apoptosis. J Exp Med.

[46] Ren D, Tu HC, Kim H, Wang GX, Bean GR, Takeuchi O, Jeffers JR, Zambetti GP, Hsieh JJ, Cheng EH (2010). BID, BIM, and PUMA are essential for activation of the BAX- and BAK-dependent cell death program. Science.

[47] Merino D, Khaw SL, Glaser SP, Anderson DJ, Belmont LD, Wong C, Yue P, Robati M, Phipson B, Fairlie WD, Lee EF, Campbell KJ, Vandenberg CJ, Cory S, Roberts AW, Ludlam MJ (2012). Bcl-2, Bcl-x(L), and Bcl-w are not equivalent targets of ABT-737 and navitoclax (ABT-263) in lymphoid and leukemic cells. Blood.

[48] Al-Harbi S, Hill BT, Mazumder S, Singh K, Devecchio J, Choudhary G, Rybicki LA, Kalaycio M, Maciejewski JP, Houghton JA, Almasan A (2011). An antiapoptotic BCL-2 family expression index predicts the response of chronic lymphocytic leukemia to ABT-737. Blood.

[49] Tahir SK, Wass J, Joseph MK, Devanarayan V, Hessler P, Zhang H, Elmore SW, Kroeger PE, Tse C, Rosenberg SH, Anderson MG (2010). Identification of expression signatures predictive of sensitivity to the Bcl-2 family member inhibitor ABT-263 in small cell lung carcinoma and leukemia/lymphoma cell lines. Mol Cancer Ther.

[50] Balmanno K, Cook SJ (2009). Tumour cell survival signalling by the ERK1/2 pathway. Cell Death Differ.

[51] Ohren JF, Chen H, Pavlovsky A, Whitehead C, Zhang E, Kuffa P, Yan C, McConnell P, Spessard C, Banotai C, Mueller WT, Delaney A, Omer C, Sebolt-Leopold J, Dudley DT, Leung IK (2004). Structures of human MAP kinase kinase 1 (MEK1) and MEK2 describe novel noncompetitive kinase inhibition. Nat Struct Mol Biol.

[52] Ohshiro K, Rayala SK, Williams MD, Kumar R, El-Naggar AK (2006). Biological role of estrogen receptor beta in salivary gland adenocarcinoma cells. Clin Cancer Res.

[53] Cragg MS, Jansen ES, Cook M, Harris C, Strasser A, Scott CL (2008). Treatment of B-RAF mutant human tumor cells with a MEK inhibitor requires Bim and is enhanced by a BH3 mimetic. J Clin Invest.

[54] Wickenden JA, Jin H, Johnson M, Gillings AS, Newson C, Austin M, Chell SD, Balmanno K, Pritchard CA, Cook SJ (2008). Colorectal cancer cells with the BRAF(V600E) mutation are addicted to the ERK1/2 pathway for growth factor-independent survival and repression of BIM. Oncogene.

[55] Gillings AS, Balmanno K, Wiggins CM, Johnson M, Cook SJ (2009). Apoptosis and autophagy: BIM as a mediator of tumour cell death in response to oncogene-targeted therapeutics. FEBS J.

