# Supplementary material for: Targeting ERK1/2-bim signaling cascades by BH3-mimetic ABT-737 as an alternative therapeutic strategy for oral cancer
Source: Oncotarget. 2015 Oct 2;6(34):35667–83. doi: 10.18632/oncotarget.5523 (PMC4742133; doi:10.18632/oncotarget.5523)
Supplement: Supplementary file 1 [file oncotarget-06-35667-s001.pdf]

SUPPLEMENTARY FIGURE

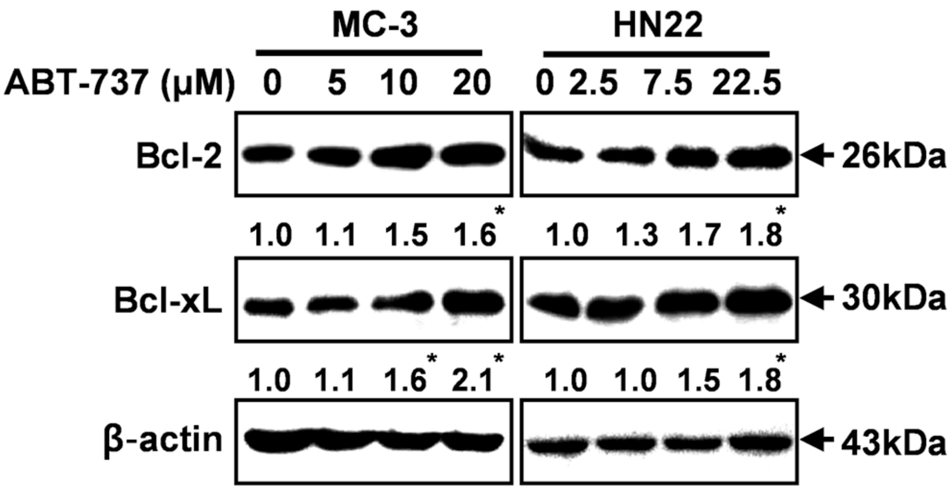

Supplementary Figure S1: Effect of ABT-737 on the expression of anti-apoptotic proteins Bcl-2 and Bcl-xL. MC-3 and HN22 cells were treated with or without ABT-737 for 24 hr, total cellular protein was prepared, and the protein levels of Bcl-2 and Bcl-xL were evaluated by Western blot analysis. β-actin was used as an internal control. The results are shown as the mean ± SD from three independent experiments. *$P < 0.05$, **$P < 0.01$, and ***$P < 0.001$.
